# Structural basis for suppression of hypernegative DNA supercoiling by *E. coli* topoisomerase I

**DOI:** 10.1093/nar/gkv1073

**Published:** 2015-10-20

**Authors:** Kemin Tan, Qingxuan Zhou, Bokun Cheng, Zhongtao Zhang, Andrzej Joachimiak, Yuk-Ching Tse-Dinh

**Affiliations:** 1 Structural Biology Center, Biosciences, Argonne National Laboratory, 9700 S. Cass Avenue, Argonne, IL 60439, USA; 2 Department of Chemistry and Biochemistry, Florida International University, 11200 SW 8 St, Miami, FL 33199, USA; 3 Department of Biochemistry and Molecular Biology, Basic Science Building, New York Medical College, Valhalla, NY 10595, USA; 4 Biomolecular Sciences Institute, Florida International University, 11200 SW 8 St, Miami, FL 33199, USA

## Abstract

*Escherichia coli* topoisomerase I has an essential function in preventing hypernegative supercoiling of DNA. A full length structure of *E. coli* topoisomerase I reported here shows how the C-terminal domains bind single-stranded DNA (ssDNA) to recognize the accumulation of negative supercoils in duplex DNA. These C-terminal domains of *E. coli* topoisomerase I are known to interact with RNA polymerase, and two flexible linkers within the C-terminal domains may assist in the movement of the ssDNA for the rapid removal of transcription driven negative supercoils. The structure has also unveiled for the first time how the 4-Cys zinc ribbon domain and zinc ribbon-like domain bind ssDNA with primarily π-stacking interactions. This novel structure, in combination with new biochemical data, provides important insights into the mechanism of genome regulation by type IA topoisomerases that is essential for life, as well as the structures of homologous type IA TOP3α and TOP3β from higher eukaryotes that also have multiple 4-Cys zinc ribbon domains required for their physiological functions.

## INTRODUCTION

The level of DNA supercoiling can have highly significant implications for vital cellular processes, and topoisomerases are therefore required in every organism to prevent accumulation of excessive supercoiling (1). Hypernegative supercoiling has been shown to lead to RNA–DNA hybrid (R-loop) stabilization (2,3). Accumulation of R-loops could inhibit transcription and replication, resulting in genomic instability (4–6). *Escherichia coli* topoisomerase I (EcTOP1) encoded by the *topA* gene is the prototype type IA topoisomerase required for preventing excess negative DNA supercoiling. It has an important role in removing transcription-induced negative supercoiling behind the RNA polymerase complex (7,8). R-loop accumulation would be an expected consequence of the loss of topoisomerase I function. The effect of *topA* deletion is more severe when RNAse H activity is depleted (9). There are also likely to be additional consequences from the loss of topoisomerase I function that would affect bacterial cell viability, as overexpression of *rnhA* did not always reverse the lethal effect of *topA* deletion (9).

Type IA topoisomerases act on a single-stranded region of DNA to initiate change in DNA topology by creating a break on the G-strand of DNA (10). A structure of the N-terminal domains of EcTOP1 (known collectively as TOP67) that form the covalent intermediate with the cleaved G-strand DNA has been previously determined (11,12). However, the N-terminal domains alone cannot catalyze the removal of negative supercoiling. The C-terminal domains of EcTOP1 are essential for the enzyme to have the capability of removing negative supercoils from DNA rapidly in a processive mechanism (13). This rapid relaxation of negative DNA supercoiling by EcTOP1 behind the RNA polymerase complex is critical for prevention of hypernegative supercoiling at highly transcribed loci. These highly transcribed loci include the rRNA genes (14) as well as the heat shock and other global response genes that are needed for survival following a stress challenge (15).

EcTOP1 as well as type IA topoisomerases in higher eukaryotes, including human type IA topoisomerases TOP3α and TOP3β, have three or more tetracysteine zinc-binding motifs in the C-terminal region that have been predicted to be part of 4-Cys zinc ribbon domains (PF01396, zf-C4_Topoisom). This protein fold found in type IA topoisomerases is related to other transcription regulators (16). Experiments have shown that there were three tightly bound Zn(II) ions present in each EcTOP1 molecule corresponding to the three 4-Cys zinc ribbon domains (17). Removal of Zn(II) (18) or mutation of cysteine residues (19) resulted in loss of the enzyme activity for removal of negative supercoils. Nevertheless, the structure of these zinc-binding motifs and how they enable the rapid relaxation of negative supercoils by EcTOP1 have remained elusive. Moreover, these type IA topoisomerase C-terminal domains also participate in protein–protein interactions important for their physiological functions, including the direct interaction between EcTOP1 and RNA polymerase beta’ subunit during transcription elongation to prevent hypernegative supercoiling (20). The C-terminal domains of Drosophila topoisomerase IIIα have been shown to be essential for double Holliday junction resolution (21). Drosophila topoisomerase IIIβ preferentially cleaves R-loops or D-loops in plasmid (22). Human topoisomerase IIIβ has also been shown to interact with the TDRD3 protein to prevent R-loop accumulation in chromatin (23).

Here, we present for the first time the structure of completely active full-length EcTOP1 in complex with single-stranded DNA (ssDNA). This provides significant new structural information on the essential C-terminal domains of type IA topoisomerases, including homologous TOP3α and TOP3β in humans. The interactions between the type IA topoisomerase C-terminal domains and ssDNA are critical for these enzymes to recognize hypernegatively supercoiled DNA as the preferred catalytic substrate. Furthermore, the C-terminal domains’ interaction with the N-terminal domains and the flexible linkers between the C-terminal domains observed in this new structure could promote the movement of the bound DNA for passage through the DNA break in order to rapidly catalyze the relaxation of negatively supercoiled DNA. Two models of how bacterial topoisomerase I could interact with ssDNA regions in partially unwound duplex DNA for removal of negative supercoils are considered here based on the new structural information. Additional biochemical data support the model in which the enzyme interacts with both the G-strand and T-strand of underwound ssDNA.

## MATERIALS AND METHODS

### Protein cloning, expression and purification

EcTOP1 was expressed from a recombinant plasmid pLIC–EcTOP in the *E. coli* BL21 STAR (DE3) strain (Invitrogen). The pLIC–EcTOP plasmid was produced by placing the EcTOP1 coding sequence into the pLIC–HK vector that allows T7 RNA polymerase-dependent expression of EcTOP1 along with a tobacco etch virus (TEV) protease-cleavable N-terminal His6 affinity tag (24). Cells were grown at 37°C and recombinant protein expression was induced by the addition of 1 mM IPTG to exponential phase cells in LB culture. After 3 h of induction, the cells were collected and subjected to freeze-thaw lysis in buffer (20 mM NaH_2_PO_4_, 0.5 M NaCl, 20 mM imidazole, 1 mg/ml lysozyme, 2.5 mM TCEP, pH 7.4). The recombinant protein molecules in the soluble lysate were allowed to bind to Ni Sepharose 6 Fast Flow (GE Healthcare) before being packed into a column. After extensive washing with buffer (20 mM NaH_2_PO_4_, 0.5 M NaCl, 20 mM imidazole, 2.5 mM TCEP, pH 7.4), the topoisomerase protein was eluted with buffer (20 mM NaH_2_PO_4_, 0.5 M NaCl, 500 mM imidazole, 2.5 mM TCEP, pH 7.4), cleaved with TEV protease, and passed through the Ni Sepharose again to remove the His6 tag. Additional purification was achieved using a ssDNA cellulose column (Sigma). The topoisomerase was eluted with an increasing concentration gradient of KCl. Final purification was carried out with an S300 size exclusion chromatography column as described previously (11).

Oligonucleotides used in site-directed mutagenesis of EcTOP1 are listed in Supplementary Table S1. The Q5 site-directed mutagenesis kit (New England BioLabs) was used to create the F616L and I701term mutants. The Quikchange site-directed mutagenesis procedures were used for the F616E and R189A mutants.

### Oligonucleotide substrate for crystallization

A partial duplex oligonucleotide substrate was produced by hybridization of a 29 base oligonucleotide (O29) 5′-GCTAAACCTGAAAGATTATGCGATTTGGG-3′ to a 20 base oligonucleotide (O20) 5′-CATAATCTTTCAGGTTTAGC-3′. The hybrid DNA O29-O20 was used for co-crystallization with EcTOP1. Since only the ssDNA corresponding to the last 11 bases of oligonucleotide O29 is visible in the crystal structure, a dozen of EcTOP1/DNA complex crystals were recovered from crystallization drops to verify the nature of bound DNA. The crystals were washed in crystallization buffer (0.125 M ammonium sulfate, 0.1 M MES pH 6.0, 1 mM zinc sulfate, and 19% polyethylene glycol monomethyl ether 5000) and dissolved in TE buffer (10 mM Tris pH 8, 1 mM EDTA). The oligonucleotide(s) present were labeled on the 5′-end with ^32^P using γ^32^P-ATP and T4 polynucleotide kinase, and analyzed by electrophoresis in 15% sequencing gel against ^32^P-labeled O20 and O29.

### Protein crystallization

For crystallization, EcTOP1 was concentrated to 38 mg/ml (∼0.39 mM). The protein was then mixed with oligonucleotide O29-O20 in a 1:1 molar ratio and the mixture was incubated on ice for 2 h. Hanging drop vapor diffusion setup was used for the crystallization with each drop containing 1 μl of the protein/DNA mixture and 1 μl crystallization solution. Diffraction quality crystals appeared under condition: 0.125 M ammonium sulfate, 0.1 M MES pH 6.0, 1 mM zinc sulfate, and 19% polyethylene glycol monomethyl ether 5000 at 24°C. Prior to data collection, the crystals were treated with cryoprotectant (25% glycerol) and cryocooled directly in liquid nitrogen.

### X-ray diffraction and structure determination

X-ray diffraction data were collected at 100 K from EcTOP1/DNA crystals at the Structural Biology Center 19-ID beamline at the Advanced Photon Source at Argonne National Laboratory using the program SBCcollect (25). The intensities of each data set were integrated, scaled and merged with the HKL3000 program suite (26). Diffraction extends to 2.90 Å resolution (Table 1) in the best data set. For structure determination using the molecular replacement (MR) method (27), two models were used as search templates: the Apo form of N-terminal domains of EcTOP1 mutant D111N (PDB entry: 3PWT) and its complex with ssDNA (PDB entry: 3PX7) (11). Rotation and translation function searches, rigid body refinement and restrained refinements suggested that the Apo form of EcTOP1 was a much better starting model than the mutant complex with ssDNA. Some results from MR with wild-type EcTOP1 as a searching template are shown in Table 1. The programs used for the MR and subsequent refinements are incorporated in the HKL3000 suite (26). After restrained refinement, difference maps clearly showed a rotation of N-terminal domain 2 (D2) in relation to its starting model. Additional densities for a number of β-sheets near N-terminal domain 4 (D4) were observed, suggesting that they were from the C-terminal domains. One cycle of auto-model rebuilding and refinement was performed with programs in HKL3000 (26), resulting in an improved model with R/R_free_ = 32.4/41.1% and FOM = 66.4.

**Table 1.** Data collection and refinement statistics

Data collection	EcTOP1/ssDNA (1)	EcTOP1/ssDNA (2)
Space group	P2_1_	P2_1_
Unit Cell dimensions		
a, b, c (Å)	85.61, 80.30, 97.48	85.56, 80.35, 97.96
α, β, γ (°)	90, 91.26, 90	90, 91.95, 90
Resolution (Å)*	37.80–2.90	41.90–3.62
	(2.95–2.90)	(3.68–3.62)
R_merge_	0.064 (0.676)	0.167 (0.623)
I/σ(I)	23.5 (2.0)	21.8 (3.4)
Completeness (%)	99.6 (100.0)	99.3 (98.0)
Redundancy	3.5 (3.6)	6.6 (6.3)

***Refinement***		
Resolution (Å)	2.90	
No. reflections	29 508	
R_work_/R_free_	0.211/0.261	
No.of atoms		
Protein	6159	
DNA	252	
Water/Others	38/31	
B-factors (Å^2^)		
Protein	93.29	
DNA	139.3	
Water/Others	66.48/105.3	
R.m.s deviation		
Bond length (Å)	0.002	
Bond angle (°)	0.526	
PDB code	4RUL	

EcTOP1, *Escherichia coli* topoisomerase I; ssDNA, single-stranded DNA; PDB, Protein Data Bank; RMS, root-mean-square.

Note: EcTOP1/ssDNA crystal 1 was used for data collection at 12.66 KeV for structure determination. EcTOP1/ssDNA crystal 2 was used for data collection at 9.67 KeV (Zn absorption edge) to verify Zn sites.

*Highest-resolution shell is shown in parenthesis.

Model re-building of the N-terminal domains and the building of the C-terminal domains were subsequently performed manually by using the program Coot (28). The C-terminal domains' region starts from three 4-Cys zinc ribbon domains. The difference electron density maps were informative enough to trace most of the main chain and register the amino acid sequence for the three 4-Cys zinc ribbon domains. After several alternative cycles of model building and refinement, the densities for one ssDNA, starting from a 3′-OH end became apparent. One DNA strand based on the sequence of oligo O29 was therefore built into model. The electron densities for the approximately last one hundred residues of the C-terminal domains were relatively poor, with only two partial β-sheets visible at low contour levels (<1σ). Since the solution structure of this region has been reported (PDB entry: 1YUA) (29), the first model of the NMR structure was used for the interpretation of electron density and model building in this region. Remarkably, the NMR model, namely the two β-sheets and two connecting α-helices could be moved into densities without major adjustment. However, the densities for some side chains and two connecting loops were missing or disordered and these side chains and loops were not included in the final model.

X-ray fluorescence spectra were measured from crystals and showed the presence of zinc in the protein crystals (data not shown). Since 1 mM zinc sulfate was present in the crystallization buffer and the resolution limit of the X-ray diffraction data was 2.9 Å, one more X-ray diffraction data set was collected at zinc absorption peak (9.6688 KeV) to pinpoint anomalous scatter zinc sites inside crystal structure. The EcTOP1 structure was then refined with the low-energy data set and anomalous difference maps were subsequently calculated (Table 1). The maps confirmed the presence of three zinc atoms at the metal-binding sites of the three 4-Cys Zn ribbon domains. An additional, fourth minor zinc site was found associated with an exposed H566 residue.

Final refinements (against 2.90 Å date set) were carried out with the program Phenix.refine and included TLS refinement (Table 1) (30). Restrains for Zn-S bond distance (2.34 ± 0.05 Å) and S-Zn-S bond angles (109.5 ± 10°) were applied and X-ray/stereochemistry weight was optimized (Table 1). Structural validation was performed using the program MolProbity (31) (Table 1).

### Biochemical analysis of EcTOP1 site-directed mutants

Relaxation activity of wild-type and mutant EcTOP1 was compared after serial dilutions using supercoiled pBAD/Thio plasmid DNA as a substrate in a buffer containing 20 mM Tris-HCl pH 8, 50 mM NaCl, 0.1 mg/ml gelatin and either 6 mM or 11 mM MgCl_2_ as indicated. After incubation at 37°C for 30 min, relaxation reaction products were analyzed by agarose gel electrophoresis (32). DNA cleavage and religation activities were assayed using a 39-base oligonucleotide substrate labeled with ^32^P on the 5′ end (5′-GATTATGCAATGCGCTTTGGGCAACCAAGAGAGCATAAC-3′) as described previously (32). Oligonucleotide substrate and cleavage products were separated by electrophoresis in a 15% sequencing gel and visualized by Phosphorimager.

### *In vivo* complementation of temperature sensitive topA mutation

Complementation of the temperature sensitive *topA* function in *E. coli* AS17 (F^−^ *topA*17(am) pLL1(*Tet supD43,74*)) (33) for growth at the non-permissive temperature of 42°C by wild-type or mutant pLIC–EcTOP was assayed by first growing the transformants overnight at 30°C in LB broth containing tetracycline (15 μg/ml) and kanamycin (50 μg/ml). Serial dilutions of the overnight cultures were spotted on LB plates with kanamycin and incubated at 30°C or 42°C.

### Nuclease footprinting of topoisomerase I binding

The production of G116S mutant of EcTOP1 was reported earlier (34). This mutant enzyme that forms a stabilized irreversible covalent complex after DNA cleavage was used to monitor topoisomerase I binding. A bubble-substrate mimicking underwound DNA was designed to have a single topoisomerase cleavage site in the single-stranded region of the top strand (Figure 9). The bottom strand has no cytosine base in the single-stranded region that can provide specific interaction for cleavage in the single-stranded region (24), so it can only act as the T-strand. The top or bottom strand was labeled at the 5′-end with T4 polynucleotide kinase and γ-32P-ATP before hybridization with the other strand to form the bubble substrate. Incubation with EcTOP1–G116S was carried out in 40mM Tris-HCl, 0.1 mg/ml BSA, 5 mM MgCl_2_ for 1 h at 37°C before digestion with 5 ng of DNase I or 7.5 pg of Micrococal nuclease (both from New England BioLabs) for 2 min and 1 min, respectively, at 37°C. Nuclease digestion was terminated by addition of 50 mM EDTA. The reaction products were analyzed by electrophoresis in a 15% sequencing gel, followed by Phosphorimager analysis.

## RESULTS

### Full-length EcTOP1 structure

**Figure 1.**
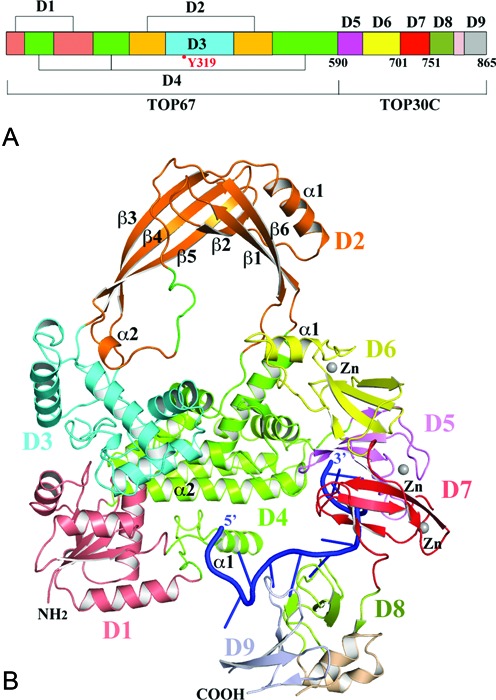
Structure of full-length *E. coli* topoisomerase I (EcTOP1) in complex with single-stranded DNA (ssDNA). (**A**) Domain arrangement of *E. coli* topoisomerase I. Between D8 and D9, there is a helical hairpin. (**B**) A ribbon diagram of full-length EcTOP1 in complex with ssDNA. Full-length EcTOP1 includes four N-terminal domains: D1 (deep salmon), D2 (orange), D3 (cyan) and D4 (green); and five C-terminal domains: D5 (pink), D6 (yellow), D7 (red), D8 (lime) and D9 (grey). The helical hairpin between D8 and D9 is colored in wheat. A ssDNA that binds to the C-terminal domains is colored in blue. Each Zn(II) is represented as a gray sphere. The secondary structures of D2 and part of D4 and D6 are labeled for discussion purposes. A part of the loop (colored in green) between α2 and β6 of D2 includes a charged and conserved sequence of R442KGDEDR, which is highly flexible and was not observed in earlier TOP67 structures. Figures 1B, 2, 4 and 7A are prepared with the program PyMOL (http://www.PyMOL.org).

The structure of full length EcTOP1 with bound DNA was determined at 2.90 Å resolution using MR with a TOP67 structure (PDB entry: 3PWT) as the search template. The full-length structure was completed with alternative cycles of manual model building and refinement (Table 1). There is one full-length EcTOP1 molecule in the asymmetric unit (Figure 1). No stable quaternary assembly is predicted based on PISA (Protein, Interface, Structures and Assemblies) analysis (35). This result is consistent with the observation that EcTOP1 is a monomer in solution.

EcTOP1 has been previously described as a protein consisting of three domains, a 67 kDa N-terminal domain (TOP67), a zinc-binding domain and a 14 kDa C-terminal DNA-binding domain (20,29,36–38). For the convenience of the following presentation and discussion of the full-length EcTOP1/DNA complex structure, the C-terminal portion zinc-binding domain and 14 kDa C-terminal DNA-binding domain together will be referred to as TOP30C. TOP67 consists of a total of four domains (D1–D4) (Figure 1). As revealed in this study, TOP30C actually contains three 4-Cys zinc ribbon domains (D5–D7) and two zinc ribbon-like domains (D8–D9). The solution structures of D8 and D9 connected through a helical hairpin (Figure 1) have been reported previously (29).

In the structure of full-length EcTOP1 in complex with DNA, the ssDNA segment is bound to TOP30C with its 3′-OH end primarily interacting with D5. The extended ssDNA also interacts with D7, D8 and D9 but not D6. Arginine 189 from TOP67 D4 contributes additional interactions with two phosphate groups of the backbone of the ssDNA chain. From the 3′-OH end a total of 11 nucleotides can be recognized and assigned a sequence (3′-GGGTTTAGCGT). The electron density for the ssDNA strand gradually becomes weak and smeared, particularly after its interaction with the last C-terminal domain D9. Two more sugar–phosphate repeats of the backbone of the strand are also visible and included in the model. The DNA present in the crystals was analyzed by gel electrophoresis with 5′-^32^P end labeling and compared with the original O29 and O20 oligonucleotides used for co-crystallization. We confirmed that both intact strands were present in the crystals (Supplementary Figure S1). A percentage (21%) of O29 was converted into its cleavage product (cleavage site five bases from the 3′-end of O29) by the EcTOP1 enzyme, likely after the crystals were dissolved in TE buffer for analysis.

### TOP67: new features in the full length structure

The overall arrangement of TOP67 in the full-length EcTOP1 is similar to what was observed previously in other TOP67 structures (e.g. PDB code: 1ECL) (38) (Figure 1). Pairwise alignments of D1s, D2s, D3s and D4s from two structures using secondary-structure match (SSM) (39) result in root-mean-square-deviation (RMSD) values of 0.59, 0.83, 0.44 and 0.67 Å, respectively, suggesting that the conformation of each individual N-terminal domain is conserved. The conformation of the active site of the enzyme, which includes residues D111, D113, Y319 and R321 from D1 and D3 are essentially the same (Supplementary Figure S2). However, if all four N-terminal domains were used for an alignment, the RMSD value is as high as 1.84 Å, which indicates domain-domain movements between the two structures. Therefore the presence of the C-terminal domains with bound ssDNA may have an effect on the conformation of the toroid formed by the N-terminal domains. The largest change involves D2 that rotates (∼14°) away from the N-terminal domains’ toroidal plane (Supplementary Figure S3).

A loop between the α2 helix and the β6 strand of D2, which was mostly disordered in previous TOP67 structures, can now be observed in this structure (Figure 1). The flexible portion of the loop comprising of a charged sequence, R442KGDEDR, extends into the center of the toroid hole and might have an important role in the proposed route of the passing strand in the DNA relaxation catalyzed by the enzyme. The corresponding region in other bacterial topoisomerase I sequences are also rich in charged residues (Supplementary Figure S4). In the structure of *E. coli* topoisomerase III, a similarly charged loop, R454RDEEND, is also present (40). It is possible that the structural ordering of the charged loop as well as the rotation of D2 as described above in the full-length EcTOP1 may be related to the presence of C-terminal domains and/or DNA binding to the C-terminal domains as elaborated below.

### TOP30C 4-Cys zinc ribbon domains and zinc ribbon-like domains

**Figure 2.**
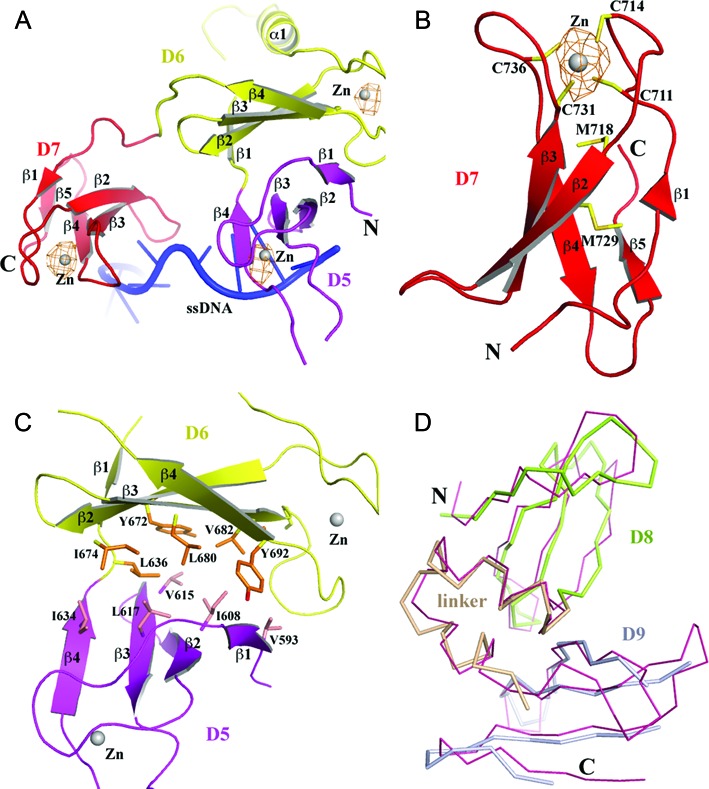
Structures of C-terminal domains. (**A**) A ribbon drawing of the first three C-terminal 4-Cys zinc ribbon domains, D5 (in pink), D6 (in yellow) and D7 (in red). All secondary structures are labeled. D6 has a unique helix (α1) between strands β1 and β2. The Zn(II) ions are drawn as gray spheres. The anomalous difference electron density map (drawn in orange mesh) around each Zn(II) is contoured at 4σ level and calculated at 4.0 Å using diffraction data collected at the Zn absorption peak. (**B**) Details of D7. The four cysteines that bind Zn(II), the conserved methionine (M718) below the Zn(II)-binding site and the hydrophobic residue (M729 in case of D7) below M718 are drawn in stick format. (**C**) The β-sheet to β-sheet packing between D5 and D6. The hydrophobic residues across their interface are drawn in stick format. (**D**) Comparison of the crystal structure and solution structure (PDB: 1YUA, in thin salmon Cα trace format) of D8 and D9 and the linker between them.

**Figure 3.**
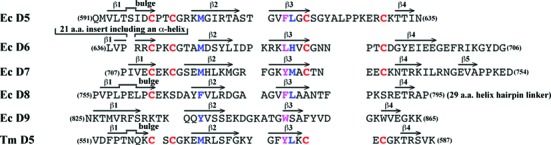
A structure-based sequence alignment of *E. coli* EcTOP1 C-terminal domains and the sole *T. maritima* TmTOP1 C-terminal domain. EcTOP1 D5 was used for pairwise structural alignments from all other individual domains using SSM. The secondary structures, β strands, are represented by arrows above appropriate sequences of each domain. The bugles on some of the lines for the β1 strands indicate the presence of one- or two-residue β-bulges on the strands. All cysteines are highlighted in red. The residues that contribute to the cores of 4-Cys zinc ribbon domains or zinc ribbon-like domains (EcTOP1 D8 and D9) are highlighted in blue. The aromatic residues from the β3 strands, which can form π–stacking interactions with nucleotides in the C-terminal domains are highlighted in magenta.

The first three TOP30C domains (D5–D7) are 4-Cys zinc ribbon domains (Figure 2A). Each domain comprises a 4-stranded antiparallel β-sheet with a Zn(II)-binding site on the top of the domain. The Zn(II) ion is coordinated by four conserved cysteines from the β1-β2 and β3-β4 loops (Figures 2B and 3). This is consistent with earlier predictions based on sequence homology and biochemical data (16,17). The presence of zinc at the metal-binding site of each domain is confirmed by anomalous difference maps calculated from diffraction data collected at the zinc absorption peak (Figure 2A–B, Table 1). The presence of the Zn(II) ions in three 4-Cys zinc ribbon domains of EcTOP1 is in contrast to the absence of Zn(II) in the 4-Cys zinc ribbon domain in the structure of *Thermotoga maritima* topoisomerase I (41). The four cysteines in the latter structure unexpectedly form two disulfide bonds instead. Beneath the Zn(II)-binding site of each domain there are a conserved methionine from the β2 strand and a hydrophobic residue from the β3 strand (Figure 3). These two residues seemingly form the core of the small 4-Cys Zn ribbon domain (Figure 2B). D5 has a one-residue β−bulge in the middle of its β1 strand (not shown in figures) while D6 has an unusual 21-amino acids insert in the middle of the first strand. A part of the insert forms a unique α-helix (α1) (Figures 1, 2A and 3), which interacts with TOP67 and is elaborated below. One unique feature of D7 is that it has an extra short β5 strand (Figure 2B).

D5 makes few contacts with D4. There are only one hydrogen bond contributed by the first residue of D5 (Q591) to D4 and a few van der Waals contacts between D5 and TOP67. However, it is D6 that forms extensive interactions with TOP67, mostly through its unique helix (α1 of D6) (Figure 1). The interactions involve TOP67's D2 and D4 and the hinge between them. A movement of the α1 helix or D6 itself against the hinge (e.g. a push or a pull) will cause the rotation of D2 in respect to D4. The change in relative orientation of two N-terminal domains could play a role in the control of the opening-closing state of the toroid hole, suggesting a possible EcTOP1 activity regulation by a TOP30C movement. Additionally, D5 and D6 adopt an unusual conformation with their β-sheets being packed against each other (Figure 2C). The interface of the two domains is rich in hydrophobic residues (Figure 2C); the movement of D5 and D6 may therefore be highly coordinated. D7 is flexibly connected to both D6 and D8. Within the linker (∼10 residues) between D6 and D7 there are two consecutive β turns, which seemingly maintain some space between the two domains. The three proline residues in the linker (P750PKEDP) between D7 and D8 may well play a similar role.

D8 and D9 domains (Figure 2D) have similar 4-Cys zinc ribbon domain folds as D5, D6 and D7, but without a Zn-binding site and the conserved methionine that is present in the β2 strand of D5, D6 and D7. This methionine is replaced by a phenylalanine (in D8) or a tyrosine (in D9) (Figure 3). The helix–hairpin linker between D8 and D9 likely helps maintain not only the fold of these two domains individually but also a stable D8–D9 conformation.

### Interaction of C-terminal domains with ssDNA

**Figure 4.**
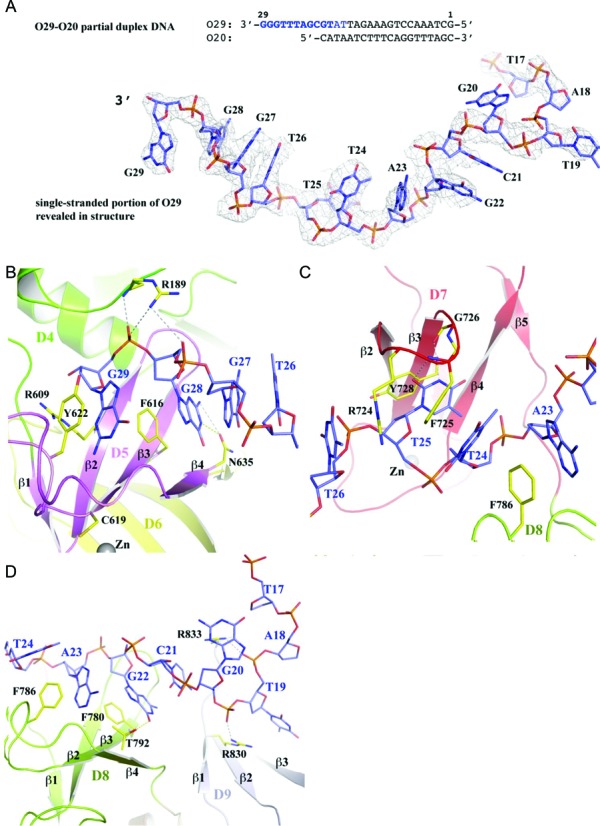
ssDNA binding to EcTOP1 C-terminal domains. (**A**) The oligo O29-O20 partial duplex used for co-crystallization with EcTOP1. The overhang of the 3′-end of O29 oligo represents a ssDNA segment that is bound to the C-terminal domains of EcTOP1. The nucleotides that were observed in the structure are highlighted in dark blue. The two nucleotides that are highlighted in light blue have disordered bases. Other parts of the oligo O29-O20 are completely disordered. The electron density drawn in gray mesh for the ssDNA segment of the DNA bound to the C-terminal domains is calculated from a weighted 2Fo-Fc map and contoured at 1σ level. (**B**) The interactions between the 3′-end of ssDNA with the first C-terminal domain D5 as well as the last N-terminal domain D4. Only one coordinating cysteine of the Zn(II), C619, is shown for the purpose of discussion. D6 colored in yellow is not involved in ssDNA binding. (**C**) The interactions between ssDNA and D7. (**D**) The interactions between ssDNA and D8 and D9.

The DNA used for co-crystallization (O29–O20) was annealed using two oligonucleotides, O29 and O20 (Figure 4A and Supplementary Figure S5). The annealing should create a 20-base pair DNA duplex with a nine nucleotide overhang at the 3′-end of O29. In the EcTOP1 structure, nine nucleotides of the overhang, two nucleotides from the predicted duplex portion and two additional phosphor-sugar moieties are visible.

The 3′-end of the ssDNA interacts with the first C-terminal domain, D5 (Figure 4B and Supplementary Figure S6). Binding primarily involves parallel π-stacking interactions between the guanine ring moieties of G29 and G28 to the aromatic side chains of the residues Y622 and F616, respectively. Residue F616 is from the β3 strand and the presence of an aromatic residue at the position is critical for ssDNA binding by TOP30C as elaborated later. Additionally, the N635 residue from the end of the β4-strand contributes two hydrogen bonds to the guanine ring of G28. Residue R189 is the only residue from TOP67 that contributes to ssDNA binding with two salt-bridges to the backbone phosphate groups of G29 and G28 and a hydrogen bond to the phosphate group of G29. This basic residue is conserved in only a subset of the bacterial topoisomerase I enzymes (Supplementary Figure S4). Substitution with alanine resulted in a small reduction (2- to 5-fold) of EcTOP1 relaxation activity (Supplementary Figure S7).

D7 binds three nucleotides, T26, T25 and T24 (Figure 4C). T25 binds to the center of D7, including parallel π-stacking with residue Y728 that is from the β3-strand and an equivalent to residue F616 of D5. The thymine ring of T25 also forms two hydrogen bonds to the main chain atoms. Additionally, residues R724 and F725 from the β2-β3 turn form a cation-π interaction with T26 and a parallel π-stacking interaction with T24, respectively.

D8 binds three nucleotides (T24, A23 and G22) as well (Figure 4D). The aromatic residue on the β3-strand, F780, contributes parallel and T-shaped π–π stackings with G22 and A23, respectively. Additional π–π stackings and one hydrogen bond are also observed between the protein and ssDNA (Figure 4D). After D8, the ssDNA appears to move away from D9. Although a part of the domain is disordered or partially disordered, the interactions involving two arginines, R830 and R833, are prominent (Figure 4D). These residues make cation-π interactions to nucleotide rings and salt bridges to backbone phosphate groups.

As mentioned earlier, D6 is not involved in ssDNA binding. Interestingly, this domain does not have an aromatic residue equivalent to F616 of D5, Y728 of D7 or F780 of D8 on the β3 strand (Figure 3), which can form π–π interactions with nucleotide bases of ssDNA. Instead, there is a leucine residue at this position of D6, which will not contribute any π-stacking interactions with ssDNA. Therefore, we propose that the primary function of D6 does not involve binding ssDNA. Since D6 rides on D5 and interacts with the hinge between D2 and D4 of TOP67 with its unique helix, it can potentially play a role in transferring the conformational change (including domain movement) of D5 to the N-terminal hinge upon D5 binding to ssDNA, subsequently regulating the opening-closing of the TOP67 toroid hole.

### Significance of the TOP30C–ssDNA interactions

**Figure 5.**
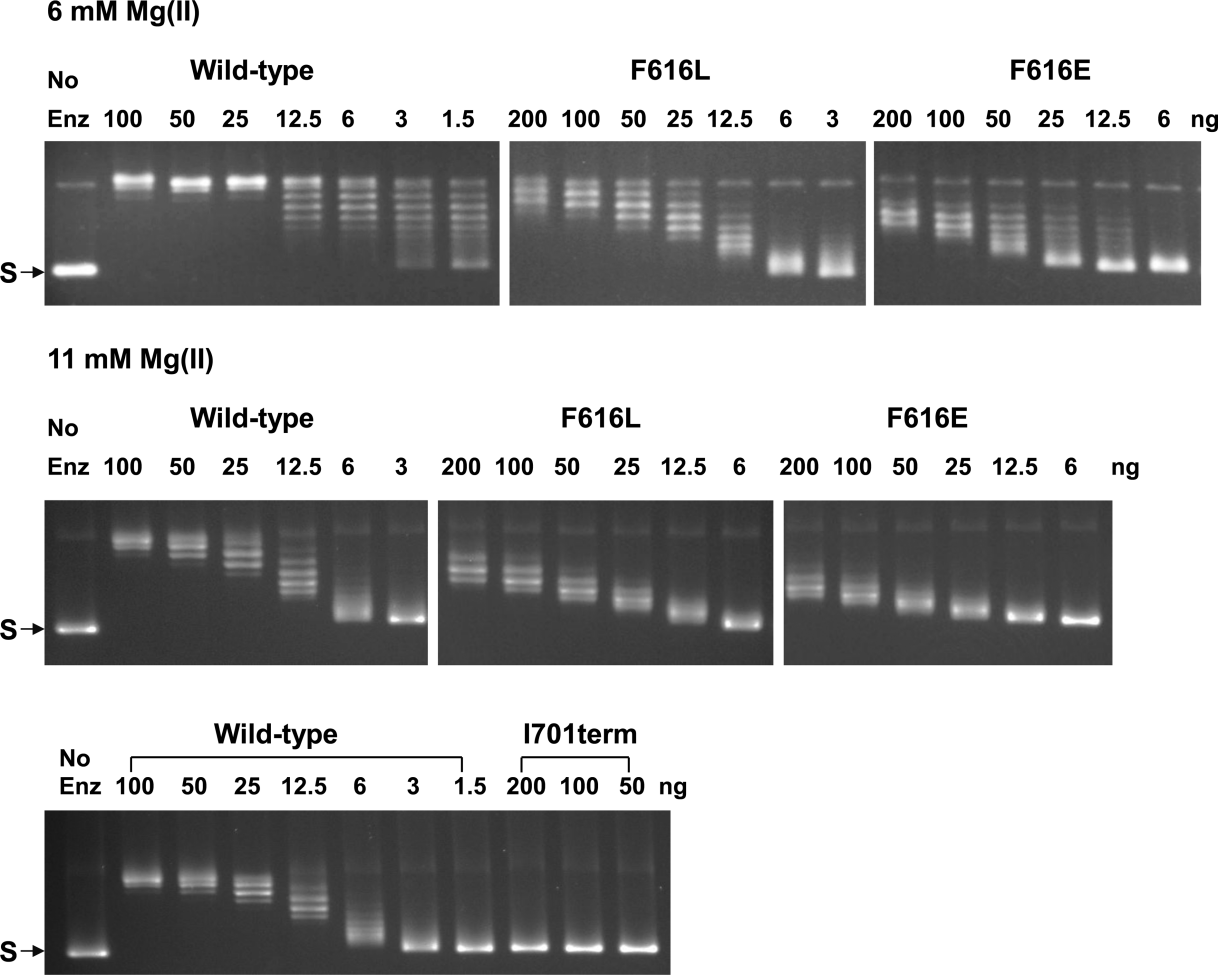
Loss of relaxation activity from C-terminal domain mutations in EcTOP1. The indicated amount of wild-type or mutant enzyme was incubated with the supercoiled plasmid DNA (S) for 30 min in reaction buffer containing either 6 mM or 11 mM MgCl_2_.

Previous studies have shown that the 85 kDa form of *E. coli* topoisomerase I missing D8 and D9 (TOP85) retained partial relaxation activity while a truncation further removing a C-terminal portion (corresponding to D6 and D7 based on the full-length structure) abolished relaxation activity completely (45). D7 is one of the four modular TOP30C domains that bind ssDNA (together with D5, D8 and D9). A truncation mutant terminating at I701 (I701term) was created to test if EcTOP1 has relaxation activity in the absence of D7. The lack of relaxation activity observed for the I701term mutant enzyme (Figure 5) showed that the interaction of D7 with ssDNA is required for the relaxation activity.

As presented above, each ssDNA-binding C-terminal domain has an aromatic residue on its β3-strand such as F616 of D5, which primarily participates in π-stacking(s) with nucleotides. To validate the importance of these stacking interactions at a distance from the active site, F616 was mutated to either a leucine (F616L) to retain hydrophobic interaction but eliminate π-stacking, or to a glutamate (F616E) to introduce a negative charge into the key ssDNA-binding site of the domain. Analysis of the relaxation activity of the F616L and F616E mutant enzymes (Figure 5) showed that when the reaction buffer contained 6 mM MgCl_2_, wild-type topoisomerase I removed negative supercoils from DNA in a processive mechanism, with full range of intermediate linking numbers observed for the reaction products from 1.5 ng of enzyme. In contrast, the F616L and F616E mutant enzymes removed negative supercoils in a distributive mechanism, with the enzyme dissociating from the DNA substrate before multiple rounds of negative supercoiling removal are accomplished. Both mutant enzymes did not fully remove the negative supercoils even with 200 ng enzyme present. When the MgCl_2_ concentration is increased to 11 mM, wild-type topoisomerase I also removed negative supercoils from DNA distributively and 4- to 8-fold lower activity was observed for the F616L mutant from the loss of the aromatic stacking interaction. The effect of the F616E mutation is more severe, with an approximately 16-fold reduction in relaxation activity from the loss of all non-polar interactions.

**Figure 6.**
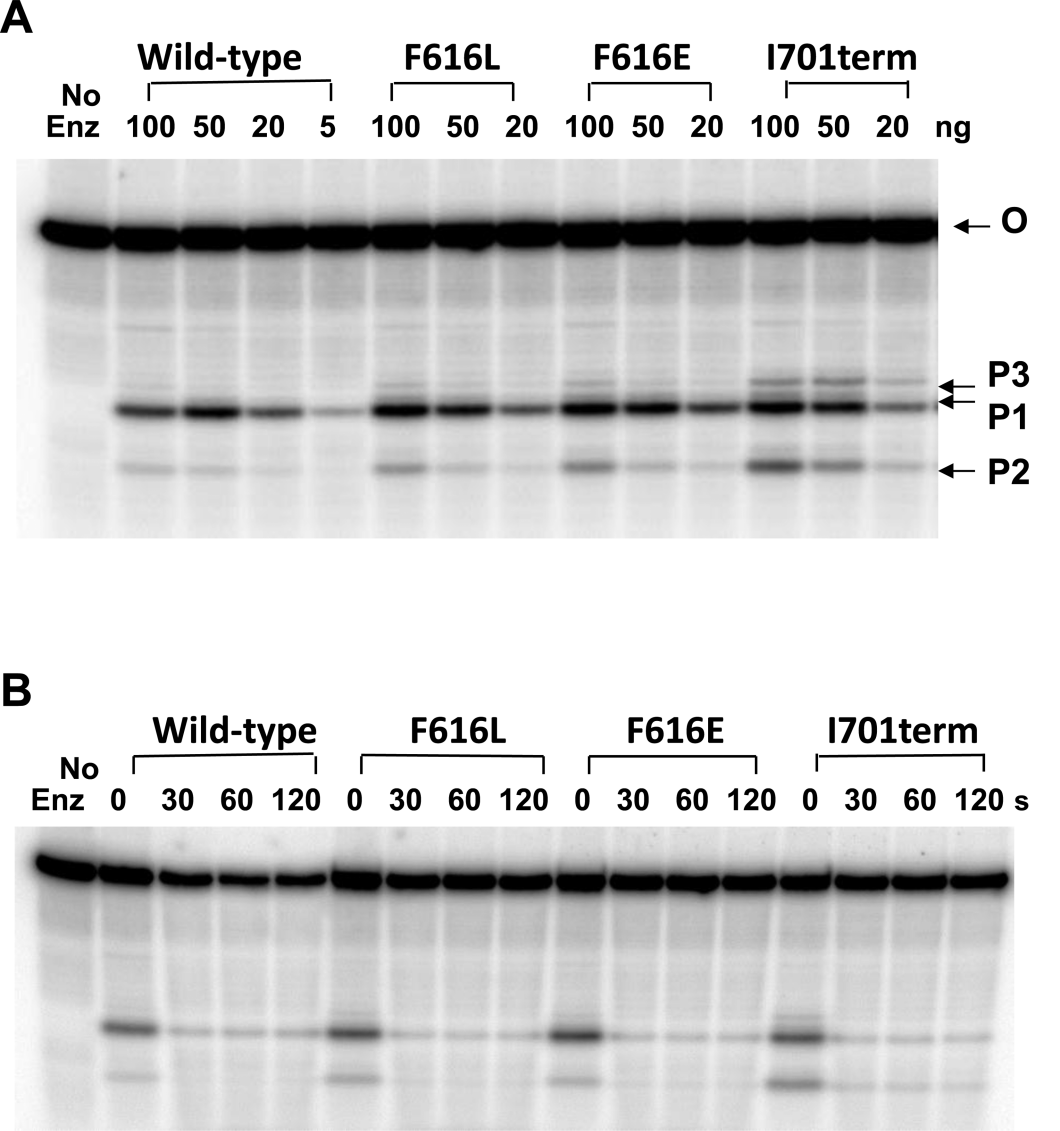
C-terminal domain mutations do not inhibit oligonucleotide cleavage and religation. The oligonucleotide substrate (O) was labeled at the 5′-end with ^32^P. (**A**) Cleavage products accumulated by the indicated amount of wild-type and mutant EcTOP1 in the absence of Mg(II). The labeled cleavage products are P1: 5′-GATTATGCAATGCGCT; P2: 5′-GATTATGCAAT; P3: 5′-GATTATGCAATGCGCTTT. (**B**) Rapid religation of cleavage products from the addition of Mg(II). Following the addition of 2 mM MgCl_2_ and 1 M NaCl to the cleavage reactions containing 100 ng of enzyme, the reactions were left on ice for the indicated time before quenching with loading buffer for sequencing gel analysis.

Further analysis showed that the deficiency in relaxation activity observed for these EcTOP1 mutants is not due to the effect of the mutations on cleavage and religation of the G-strand of DNA. Figure 6A showed that the cleavage of the oligonucleotide substrate by the F616L and F616E mutants was nearly identical to that of the wild-type with product P1 being the major cleavage product. A higher level of the alternative cleavage products P2 and P3 could be observed for the I701term mutant. Following oligonucleotide cleavage, religation driven by the addition of Mg(II) could be observed as the disappearance of the cleavage product if the enzyme is then dissociated from the religated oligonucleotide by high salt concentration. The results of such a religation assay (Figure 6B) showed that these C-terminal mutations did not inhibit religation of the G-strand.

**Figure 7.**
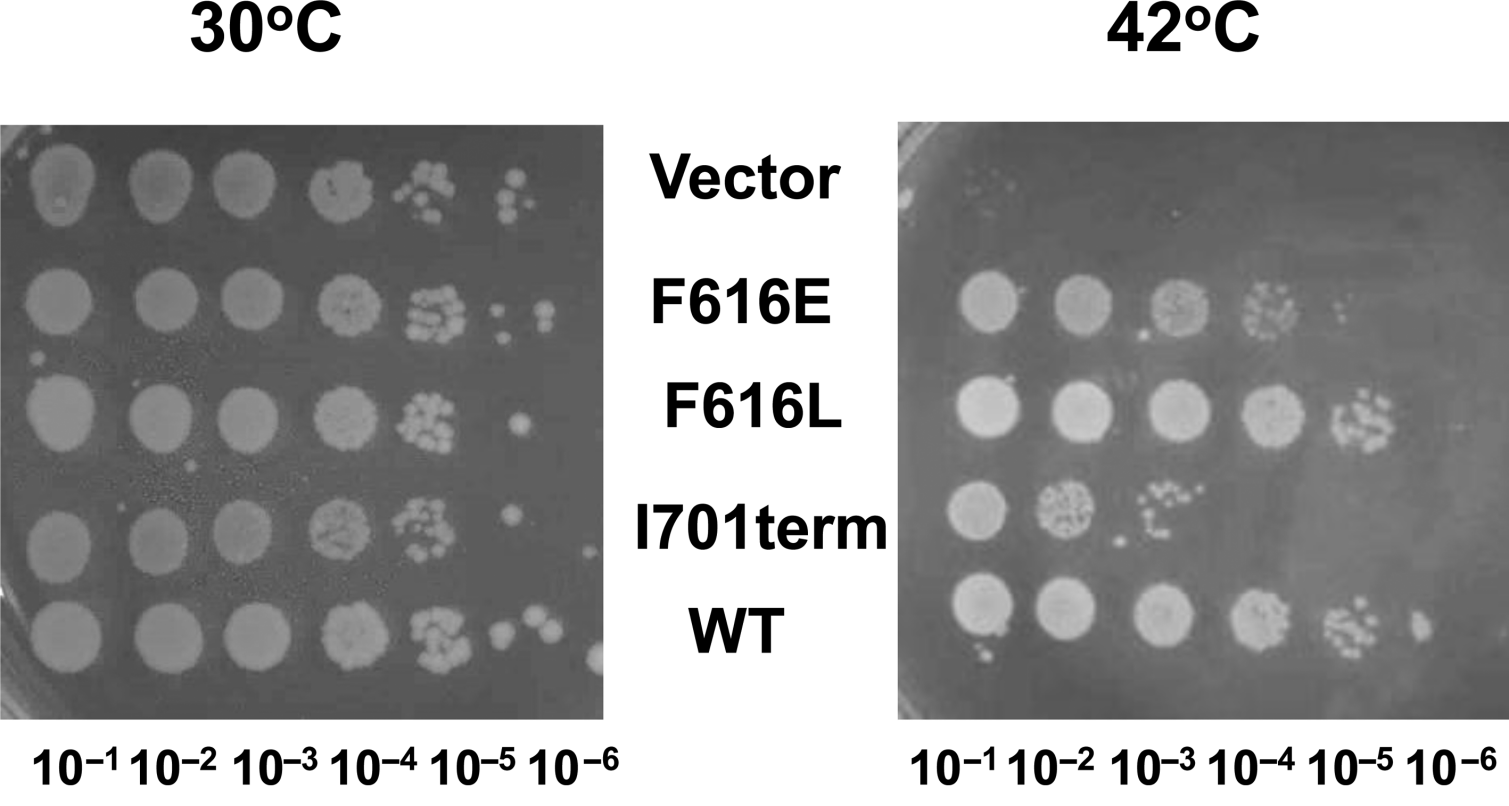
Assay of complementation of *topA* temperature sensitive function in *E. coli* AS17 by wild-type (WT) pLIC-ETOP and its mutant derivatives. Serial dilutions of overnight cultures of AS17 transformants grown at 30°C were spotted on LB plates with kanamycin, followed by incubation at either 30°C or 42°C.

The requirement of the interactions between the C-terminal domains and ssDNA is also relevant for the essential *in vivo* function of EcTOP1. *Escherichia coli* strain AS17 has a temperature sensitive *topA-*chromosomal mutation (42). The *topA* function at the non-permissive temperature can be complemented by the non-induced background topoisomerase I expression from pLIC-ETOP plasmid (33). Results in Figure 7 showed that the I701term mutant derivative of pLIC–ETOP showed the greatest deficiency in complementation of the temperature sensitive chromosomal *topA* in AS17 for growth at 42°C while the partial activity from the F616L mutant was more effective in complementation than the F616E mutant. These results are consistent with the relative severity in loss of relaxation activity from the C-terminal mutations examined.

### Testing of models by nuclease footprinting

**Figure 8.**
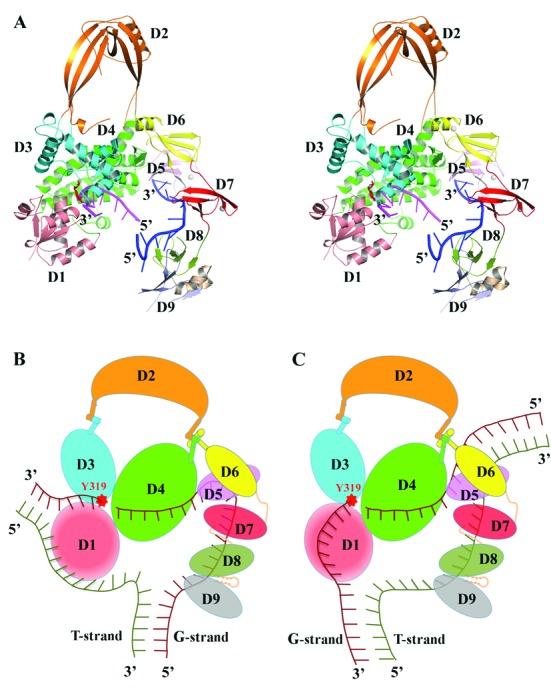
Models of EcTOP1 in interaction with ssDNAs and its implications for positioning of unwound duplex DNA. (**A**) Stereo view of a ribbon diagram of full-length EcTOP1 with two ssDNA segments bound at N- and C-terminal domains. The model was created by combining the N-terminal domains/ssDNA structure (PDB code: 3PX7) and the structure reported in this study as described in the text. (**B**) A single chain and (**C**) a double chain model of ssDNA chain(s) binding to EcTOP1 for DNA relaxation. In the single chain model, the N- and C-terminal domains interact primarily with the G-strand only. In the double chain model, the N-terminal domains interact primarily with the G-strand while the C-terminal domains interact primarily with the T-strand. D1, drawn in red with a radial gradient does not bind ssDNA. The α1 (alpha1) helix on D6 is modeled as a handle, which presumably can pull or push the hinge between D2 and D4 domains for the regulation of the opening-closing state of N-terminal toroid hole.

To gain insights into the catalytic mechanism of EcTOP1 for relaxation of supercoiled DNA, a model of EcTOP1 with two ssDNA segments bound separately to the N- and C-terminal domains was built as shown in Figure 8A. To build the model, the N-terminal domains/ssDNA complex structure previously reported (PDB code: 3PX7) and the C-terminal domains/ssDNA of this new full-length structure are combined. For a smooth transition from the D4 of 3PX7 to the D5 of the full-length structure, only D4 of the two structures were used for a SSM alignment. The N-terminal domains of full-length EcTOP1 were then replaced by 3PX7. Based on the orientations of the two ssDNAs in the comprehensive model (Figure 8A), there are two possible binding modes for the ssDNA chain(s) in the relaxation of negatively supercoiled duplex DNA, a single chain model and a double chain model. In the single chain model, the enzyme interacts only with the G-strand (Figure 8B). In the double chain model (Figure 8C), the N-terminal domains of the enzyme interacts with the G-strand while the C-terminal domains interact with the T-strand. The oligonucleotides seen in the crystal structures would correspond to a portion of the ssDNA segments in the models.

**Figure 9.**
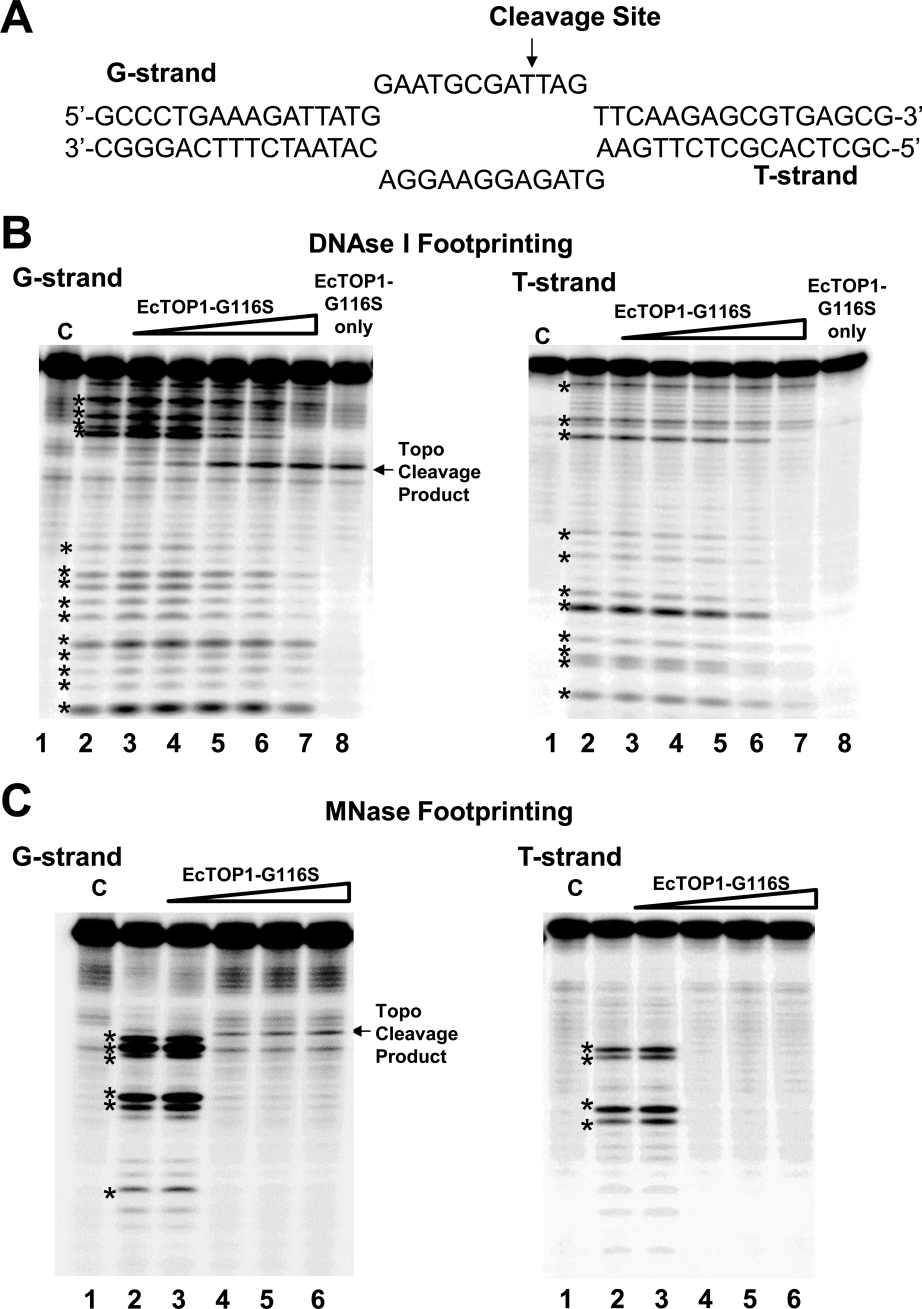
Nuclease footprinting with DNA bubble substrate. (**A**) Sequence of top (G-strand) and bottom (T-strand) of DNA bubble substrate. The topoisomerase I cleavage site is shown by arrow on the G-strand. Nuclease footprint of EcTOP1-G116S on the bubble substrate is followed by 5′ ^32^P end-labeling of either the G-strand or T-strand. (**B**) DNase I footprinting. Lane 1: Control (C), DNA only; Lane 2: Nuclease only; Lanes 3–7: 10, 20, 40, 60, 100 ng ETOP-G116S followed by nuclease; Lane 8: 100 ng of EcTOP1-G116S only. (**C**) MNase footprinting. Lane 1: Control (C), DNA only; Lane 2: Nuclease only; Lanes 3–6: 10, 20, 40, 60 ng ETOP-G116S followed by nuclease. Nuclease cleavage products are denoted by * symbol.

Nuclease footprint experiments were carried out to test these two models. The DNA bubble substrate used (Figure 9A) had a 12-nucleotide unpaired region. This length of ssDNA region was shown in previous studies to be sufficient for *E. coli* topoisomerase I to bind and relax positively supercoiled DNA that had a 12-nucleotide bubble (43). The G-strand is designed to provide a single EcTOP1 cleavage site eight nucleotides downstream of the ssDNA–dsDNA junction shown by arrow in Figure 9A, while the single-stranded region of the T-strand is not cleaved by the enzyme because the sequence does not have a cytosine in the single-stranded region that is required for a preferred EcTOP1 cleavage recognition site (Figure 9A). The G116S mutant of EcTOP1 was used for the footprinting experiment because this mutant is known to form a stabilized irreversible cleavage complex (34). Protection of the G-strand and T-strand in the hybridized bubble substrate from DNase I or micrococcal nuclease (MNase) digestion was monitored by 5′ ^32^P-end labeling of the individual strand. The DNase I cleavage took place primarily in the double-stranded region of the bubble substrate, while MNase cleaved the single-stranded region preferentially. Interaction of EcTOP1–G116S resulted in partial protection from DNase I digestion for the double-stranded region of both the G-strand and T-strand (Figure 9B) while complete protection of the ssDNA region from MNase digestion by EcTOP1–G116S can be seen for both the G-strand and T-strand (Figure 9C). These results provide further support of the double-chain model shown in Figure 8C.

## DISCUSSION

*Escherichia coli* topoisomerase I is highly efficient in removing negative supercoils because the catalytic mechanism is based on the enzyme interacting with an underwound ssDNA region present in duplex DNA. The multiple interactions between the C-terminal domains and ssDNA is a key element of the mechanism for bacterial topoisomerase I to utilize unwound duplex DNA as substrate and prevent hypernegative supercoiling *in vivo*. Without the efficient relaxation activity of topoisomerase I, hypernegative supercoiling behind the elongating RNA polymerase complex would leave the template and non-template strands of DNA in unwound single-stranded form. This hypernegative supercoiling would favor the reannealing of the RNA transcript to the template strand to form a stabilized R-loop.

Positively supercoiled DNA can also be a substrate of bacterial topoisomerase I *in vitro*, provided a single-stranded loop region is present for the enzyme to bind (43). EcTOP1 has been previously shown to cleave partial duplex DNA with structural preference of the cleavage site near the junction of ssDNA and dsDNA (44). Structures of N-terminal domains D1–D4 (TOP67) of EcTOP1 with ssDNA bound non-covalently or in covalent complex are available from previous studies (11,45). However, the N-terminal domains D1–D4 can cleave and rejoin ssDNA but cannot promote DNA strand passage through the DNA break to remove negative supercoils (37,46). Truncated EcTOP1, D1–D7 (TOP85) is capable of relaxing negatively supercoiled DNA (37), but with lower affinity for binding to ssDNA and reduced processivity for relaxation of negatively supercoiled DNA (36). The contribution of the C-terminal domains is essential for EcTOP1 to relax supercoiled DNA. It has not been known how the 4-Cys zinc ribbon domains that follow D4 might interact with the DNA substrate. The oligonucleotide O29–O20 hybrid duplex used for the co-crystallization in this study was designed originally to maintain the ssDNA binding to TOP67 and possibly extend the duplex DNA binding into the zinc ribbon domains or D5–D7 with its dsDNA segment. The 14 kDa C-terminal fragment or D8–D9 was isolated previously as a fragment with higher affinity to ssDNA than TOP67 following ssDNA affinity chromatography of partial tryptic digest of EcTOP1 (35). Therefore, another potential binding mode was a reverse of the duplex DNA orientation, in which D8–D9 binds the ssDNA portion of DNA.

The full-length EcTOP1/DNA complex structure revealed in this study has shown that four of five TOP30C domains including the two 4-Cys zinc ribbon domains (D5 and D7) and the two 4-Cys zinc ribbon-like domains of the 14 kDa C-terminal fragment (D8–D9) bind the ssDNA portion of the predicted duplex DNA, while the dsDNA part of the oligonucleotide is completely disordered without any recognizable association with EcTOP1. In the single chain model (Figure 8B), the two binding ssDNA segments shown in Figure 8A presumably represent two binding segments from a single ssDNA chain that would function as the G-strand for DNA cleavage by the enzyme to create the break through which strand passage will take place (Figure 8B). However, the length of the ssDNA spanned between the cleavage site and the 5′ upstream junction of the ssDNA–dsDNA near C-terminal D9 can be estimated to be at least 18 bases long in this single chain model. This is significantly longer than the 3–9 base distance observed previously for EcTOP1 cleavage of various DNA substrates with both ssDNA and dsDNA regions (24,44). In the alternative double chain model (Figure 8C), the two binding ssDNA segments in Figure 8A are presumed to be from two different ssDNA chains with one of the ssDNA/dsDNA junctions at or near the zinc ribbon domains D5 and D6. The distance between the cleavage site and the junction of the ssDNA/dsDNA is about 8–9 bases, which is a much better fit for the known biochemical data for cleavage by EcTOP1. The double chain model with the C-terminal domains interacting with the T-strand but not the G-strand is also consistent with the results here that show that the I701term mutant has robust cleavage and religation activity for the G-strand of DNA. The F616 mutations also did not affect cleavage and religation of the G-strand of DNA significantly. Therefore, the ssDNA bound between D5 and D9 should be a part of the passing strand of DNA in the mechanism for DNA relaxation (Figure 8C). The nuclease footprinting experiments using the bubble substrate that has a 12 base long unpaired single-stranded region provided further support of the double-chain model over the single-chain model. The cleavage site on the G-strand of this substrate is eight bases downstream from the ssDNA–dsDNA junction. Interaction of the DNA with the EcTOP1–G116S enzyme to form the stabilized covalent complex protected both strands of the unpaired region of the DNA substrate from MNase digestion as predicted by the double chain model in Figure 8C. It might be possible for the melted single-strands of DNA from the RNA polymerase transcription bubble to be bound directly by topoisomerase I N-terminal and C-terminal domains without first annealing back into double-stranded DNA. The direct interaction between topoisomerase I and RNA polymerase would facilitate such a mechanism. Further biochemical studies are needed to verify this scenario.

The interactions between ssDNA and the C-terminal domains are dominated by multiple parallel and T-shaped π–π stackings between the bases of the ssDNA and the side chains of the aromatic residues from the C-terminal domains. Noticeably, the conserved aromatic residue from the β3-strand of each zinc ribbon domain, with a Zn(II)-binding site (D5 and D7) or not (D8), is the center of these π-stacking interactions of each domain. The importance of these π-stacking interactions in the catalytic mechanism of the enzyme is clearly illustrated by the reduction in relaxation activity from the F616 mutations introduced. In addition, some basic residues, arginines or lysines, contribute a couple of cation–π interactions to the bases of ssDNA. They also form salt bridges to some backbone phosphate groups.

It should be noted that all these dominant π-stackings and cation–π interactions, together with van der Waals interactions mentioned earlier, between EcTOP1 TOP30C and ssDNA are independent of the ssDNA sequence. Only a few hydrogen bonds were observed in the ssDNA sequence and most of them appear nonspecific. For example, the residue N635, which forms two hydrogen bonds to the guanine G28, could also form at least one hydrogen bond with an adenine at the same position, and potentially with thymine or cytosine as well. The DNA sequence-independent interactions between TOP30C and ssDNA suggest that these domains can bind ssDNA of a wide range of sequences, as expected from the function of EcTOP1 in global gene expression. However, we cannot exclude any potentially preferred nucleotide sequences at given binding sites for maximal interactions between the C-terminal domains and ssDNA.

The lack of sequence specificity for ssDNA binding to EcTOP1 C-terminal domains might cause problems in the trials of the co-crystallization of EcTOP1 and ssDNA. The success of EcTOP1/O29-O20 co-crystallization was likely due to (i) the right length of ssDNA in the substrate, which covered the C-terminal domains from D5 to D9; (ii) the presence of a dsDNA segment in the substrate, which does not bind the C-terminal domains and therefore prevents random binding registration of ssDNA along the C-terminal domains. This limited the binding mode of the substrate to the C-terminal domains and helped the co-crystallization with EcTOP1.

The presence of Zn(II) has been proven to be critical to the structure and function of EcTOP1(20). In the full-length EcTOP1/O29–O20 structure, none of the three Zn(II)-binding sites in TOP30C are directly involved in ssDNA binding. D8 and D9, which do not bind zinc, bind ssDNA as well as D5 and D7. The metal binding site at the top of each zinc ribbon domain seemingly assists in stabilizing the fold of the domain and likely helps to maintain certain conformations suitable for the association and dissociation of ssDNA. In the case of D8 and D9, it may be that the helical hairpin between the two domains stabilizes their structures and maintain their ssDNA binding conformations. The removal of Zn(II) from D5, D6 and D7 would likely destabilize these domains. In the center of the C-terminal domains and with two flexible linkers to D6 and D8, D7 may play a central role in the movement of the passing ssDNA strand during the catalytic cycles of the enzyme. This is supported by the experimental data that show severe loss of relaxation activity for the I701term mutant.

In the proposed model for the DNA relaxation mechanism, the movement of the passing strand of DNA is guided by the C-terminal domains. We believe the interdomain flexibility of D7 to its preceding and succeeding domains and the coordination of the C-terminal domain movement and the opening-closing of the N-terminal toroid hole would greatly facilitate cycles of the passing strand of DNA entering and exiting the toroid hole for multiple processive cycles of removal of negative supercoils with only short pauses, as observed in single-molecule studies (47). The charged loop in domain D2 may play an active role in the entry and exit of the passing strand of DNA in and out of the toroid hole.

The N-terminal domains (D1–D4) of type IA topoisomerase enzymes, found among protein family (Pfam) (48) members of Pfam Toprim (PF01751) characterized by divalent ion interactions and Pfam Topoisom_bac (PF01131) (Supplementary Figure S8) are of high degrees of sequence conservation and presumably of high structural conservation. It is not surprising that these Pfam members have a similar catalytic function of cutting and rejoining a single strand of DNA via the 5′-phosphotyrosine intermediate. Due to the sequence diversities in their C-terminal domains, the efficiency for relaxation of negatively supercoiled DNA would then differ among the topoisomerase I and III enzymes (47). Most bacterial topoisomerase I enzymes have at least two 4-Cys zinc ribbon domains while a small number of them, such as *T. maritima* topo I, have only one zinc ribbon domain that may or may not bind Zn(II). EcTOP1 and its most closely related bacterial topoisomerase I enzymes have additional zinc ribbon-like domains without the hallmark of four zinc-binding cysteines. In the EcTOP1/ssDNA structure, it is clear that both the 4-Cys zinc ribbon domains and zinc ribbon-like domains bind ssDNA in similar fashions involving primarily multiple π-stacking interactions without direct involvement of the Zn(II)-binding sites. Zn(II) binding appears to be important for the structural stability of a zinc ribbon domain, but is not always necessary for ssDNA binding. It is possible that during evolution when a Zn(II)-binding domain acquires other structural element(s) that may help its stable folding, it may lose its Zn(II)-binding site. D8 and D9 of EcTOP1 may be examples of such an evolution of a ssDNA binding domain, in which a unique helical hairpin linker eventually clamps the two domains when they lose Zn(II)-binding cysteines.

It is noticed that a smaller subset of bacterial topoisomerase I enzymes in *Actinobacteria*, including *Mycobacterium tuberculosis*, *Mycobacterium smegmatis* (49) and *Streptomyces coelicolor* (50), do not have any recognizable 4-Cys zinc ribbon domains (Supplementary Figure S8). The C-terminal regions of bacterial and fungal topoisomerase III enzymes also do not have recognizable zinc ribbon domains. Although they are believed to utilize basic amino acid rich domains for ssDNA binding (51), the possibilities of zinc ribbon-like domains or even other types of folds in their C-terminal regions cannot be excluded.

Interestingly, topoisomerase III enzymes from higher eukaryotes evolved to have multiple 4-Cys zinc ribbon domains in their C-terminal regions (21,52,53). The structure and ssDNA interactions elucidated here for the EcTOP1 4-Cys zinc ribbon domains should be highly relevant for the structure and mechanism of human TOP3α and TOP3β that have been shown to play important roles in genomic stability and neurodevelopment, respectively (1,54,55). Human TOP3β may specifically target hypernegatively supercoiled DNA for suppression R-loops (23) in a function similar to EcTOP1. A C666R single nucleotide variant in human TOP3β has been found in individuals with autism spectrum disorders and could affect the structure of the C-terminal region (55).

Here we have reported for the first time the full-length EcTOP1 structure with ssDNA bound to its C-terminal domains. We have also for the first time characterized the interactions of the 4-Cys zinc ribbon domain and its closely related zinc ribbon-like domain with ssDNA that are likely to be important for recognition and suppression of hypernegatively supercoiled DNA. The extended knowledge of the ssDNA binding domains has improved our understanding of not only the bacterial and eukaryotic topoisomerases discussed here but also other proteins, which may contain similar zinc ribbon domains. The YrdD protein of *Enterobacteriaceae*, which seemingly contains four 4-Cys zinc ribbon domains, has been shown to have Zn(II)-dependent ssDNA binding activity and may be involved in DNA repair (56). Certain transcription factors with zinc ribbon domains, for example Brf, may utilize the zinc ribbon domains to interact with a localized unwound ssDNA region to facilitate open complex formation in transcription initiation (57).

## ACCESSION NUMBER

The atomic coordinates and structure factors of the full-length *E. coli* topoisomerase I in complex with ssDNA has been deposited in the Protein Data Bank (PDB) with accession code 4RUL.

## Supplementary Material

SUPPLEMENTARY DATA
