# Supplementary material for: Structural basis for suppression of hypernegative DNA supercoiling by E. coli topoisomerase I
Source: Nucleic Acids Res. 2015 Oct 20;43(22):11031–46. doi: 10.1093/nar/gkv1073 (PMC4678816; doi:10.1093/nar/gkv1073)
Supplement: SUPPLEMENTARY DATA [file supp_43_22_11031__index.html]

Structural basis for suppression of hypernegative DNA supercoiling by E. coli topoisomerase I — SUPPLEMENTARY DATA 

# Structural basis for suppression of hypernegative DNA supercoiling by *E. coli* topoisomerase I

## SUPPLEMENTARY DATA

- SUPPLEMENTARY DATA
- SUPPLEMENTARY DATA
- SUPPLEMENTARY DATA
