# Supplementary material for: Structural basis for suppression of hypernegative DNA supercoiling by E. coli topoisomerase I
Source: Nucleic Acids Res. 2015 Oct 20;43(22):11031–46. doi: 10.1093/nar/gkv1073 (PMC4678816; doi:10.1093/nar/gkv1073)
Supplement: SUPPLEMENTARY DATA [file supp_gkv1073_nar-02650-f-2015-File011.pdf]

Table S1. Oligonucleotides used in site-directed mutagenesis of *E. coli* topoisomerase I

F616E	Forward: 5'-CACCGGGGTAGAACTTGGCTGTTCTGG-3'<br>Reverse: 5'-CTCGCTGTGCGAATCCCC-3'
R189A	Forward: 5'- AAAGATCGCTGCTGGCCTGTCTGCCGG-3'<br>Reverse: 5'-TTCCATAGCAGCGGCGAA-3'
F616L	Forward: 5'-CAGAACAGCCAAGTAATACCCCGGTGCTCG-3'<br>Reverse: 5'-CGAGCACCGGGGTATTACTTGGCTGTTCTG-3'
I701term	Forward:<br>5'-GAAGAGGGCGAATTCCGCTAGAAAGGTTATGACGGCCCG-3'<br>Reverse:<br>5'-CGGGCCGTCATAACCTTTCTAGCGGAATTCGCCCTCTTC-3'

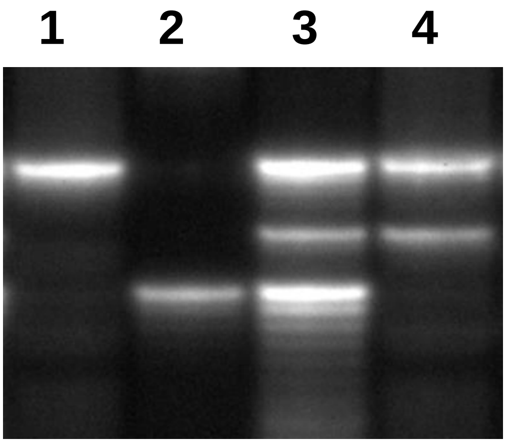

Figure S1. PhosphorImager analysis of oligonucleotides labeled by ^{32}P at 5'-end. Lane 1: Oligonucleotide O29; Lane 2: Oligonucleotide O20; Lane 3: Oligonucleotides in crystals dissolved in TE buffer (10 mM Tris HCl, pH 8, 1 mM EDTA) ; Lane 4: Oligonucleotide O29 + EcTOP1 in TE buffer incubated at 37°C for 10 min. Oligonucleotides were separated in 15% sequencing gel.

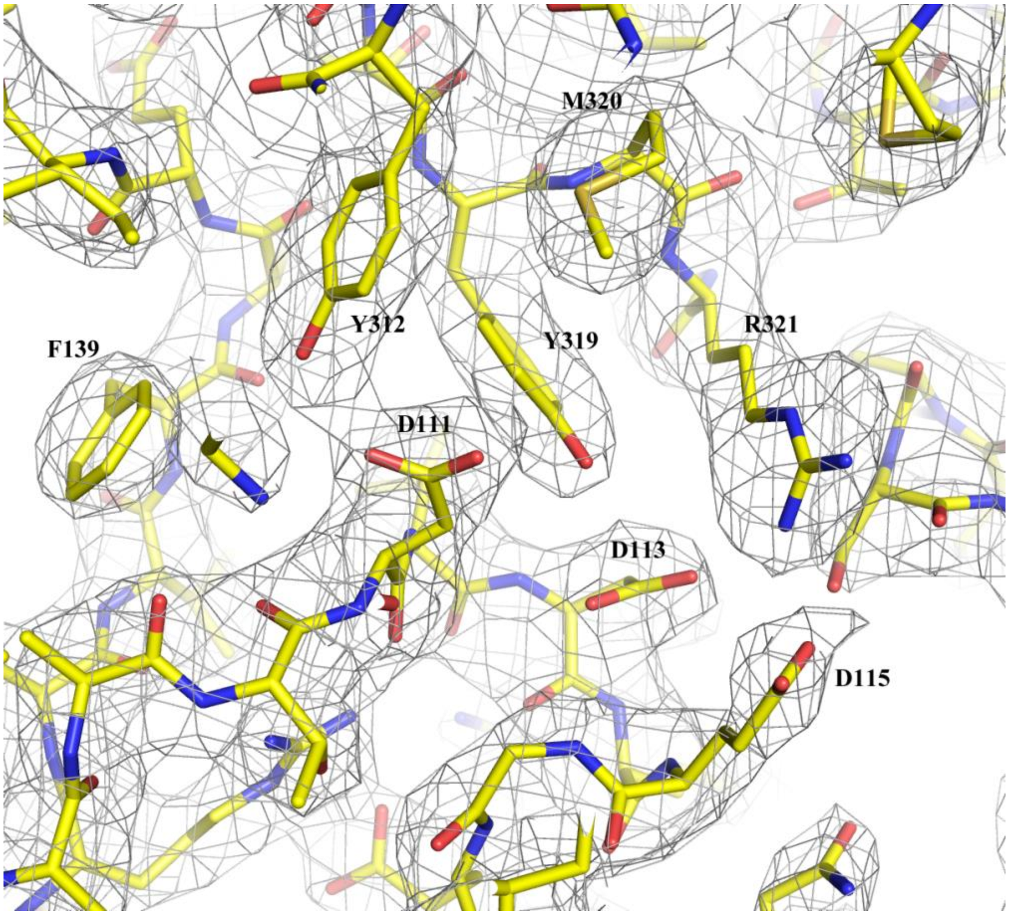

Figure S2. A stick model of the active site of the EcTOP1 structure. The residues of the active site of EcTOP1 including catalytic residue Y319 are drawn in stick format. The associated electron density of the active site is drawn in grey mesh and calculated from a weighted 2Fo-Fc map and contoured at 1σ level.

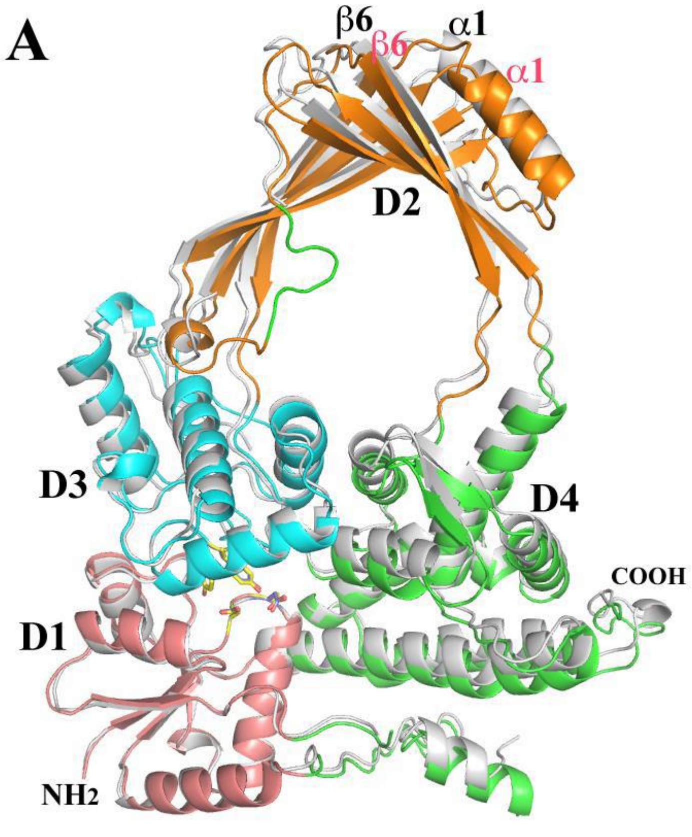

Figure S3. Structural comparison of the N-terminal domains of full-length EcTOP1 and N-terminal domains only construct. A) A ribbon diagram of a superposition of the N-terminal domains of full-length EcTOP1 and N-terminal domains only construct (PDB: 1ECL). Only the D1 domain from both structures was used for a SSM alignment calculation. The color codes for the full-length N-terminal domains are the same as those in Figure 1. The structure of 1ECL is colored in grey. Some active site residues, including D111 and D113 from D1, Y312 and Y319 from D3 and S495 from D4 are drawn in stick format. Only a few secondary structures are labeled for figure clarity and the convenience of discussion. The portion of a charged sequence, R442KGDEDR of the flexible loop on D2, is colored in green. This part of the loop is totally disordered in the structure of 1ECL. B) A view of the same superposition with the structures being rotated about minus 90 degrees around the vertical axis.

A

EcTOP1	181	PLLWKKIA**R**G	LSAGRVGSVA
HiTOP1	187	PLLWKKVA**R**G	LSAGRVQSVA
PaTOP1	186	PLLWQKIA**R**G	LSAGRVQSVA
NgTOP1	156	PLLWKKIR**R**G	LSAGRVQSPA
CjTOP1	153	PLLGQKIQ**R**G	LSAGRVQSAA
SaTOP1	152	PVLWKKVK**K**G	LSAGRVQSVA
BsTOP1	152	PILWKKVK**K**G	LSAGRVQSVA
TmTOP1	153	PVLWRNFKSN	LSAGRVQSAT
MtTOP1	180	PVLWKKVAPK	LSAGRVQSVA

B

EcTOP1	437	VMPAL	**RKGDEDR**	ILPAVNKG
HiTOP1	443	VLPQI	**GKNPEDQE**	LPSVTVS
PaTOP1	442	VLPQQ	**SKPGEDD**	VLPEMKEG
NgTOP1	424	VYEES	**SDDEESEDSKK**	LPEMSEG
CjTOP1	415	YYKVY	**GDMDKDK**	ILPNFKIG
SaTOP1	417	LYVET	**KDDSDSEKENK**	LPKLEQG
BsTOP1	417	VYVE	**GKDDQMEEKDR**	MLPDLQEG
TmTOP1	409	VW	**KTERNTGE**	FP FEEG
MtTOP1	480	V DELV	**GGEADDAERR**	LPHLTPG

Figure S4. Sequence alignment of bacterial topoisomerase I sequences (A) The region around R189 in the N-terminal D4 domain interacts with ssDNA bound to the C-terminal domains; TmTop1 and MtTOP1 do not have Zn(II) binding C-terminal domains. (B) Highly charged flexible loop at R442. Abbreviations: *Escherichia coli* (Ec), *Haemophilus influenza* (Hi), *Pseudomonas aeruginosa* (Pa), *Neisseria gonorrhoeae* (Ng), *Campylobacter jejuni*(Cj), *Staphylococcus aureus* (Sa), *Bacillus subtilis* (Bs), Thermotoga maritima (Tm), *Mycobacterium tuberculosis* (Mt).

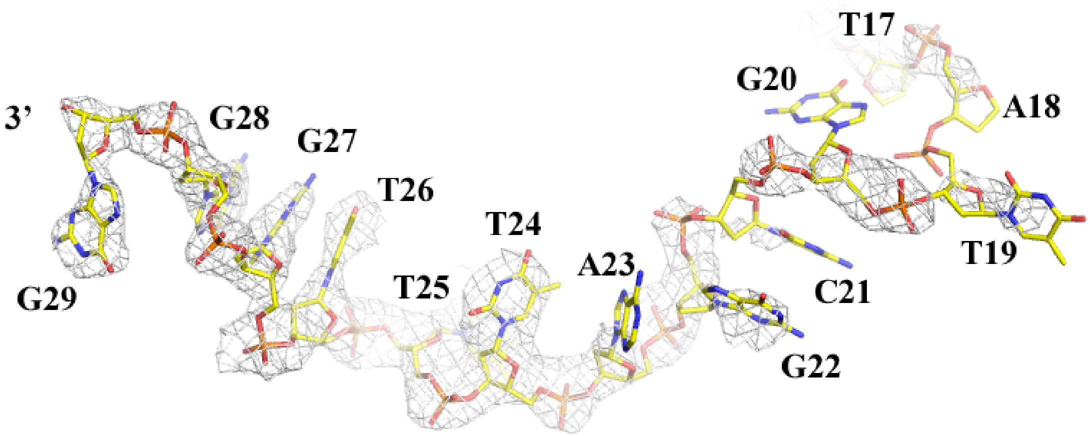

Figure S5. A stimulated annealing omit map of ssDNA. The electron density drawn in grey mesh for the ssDNA segment of the DNA bound to the C-terminal domains is calculated from a weighted 2Fo-Fc simulated annealing omit map and contoured at 1σ level.

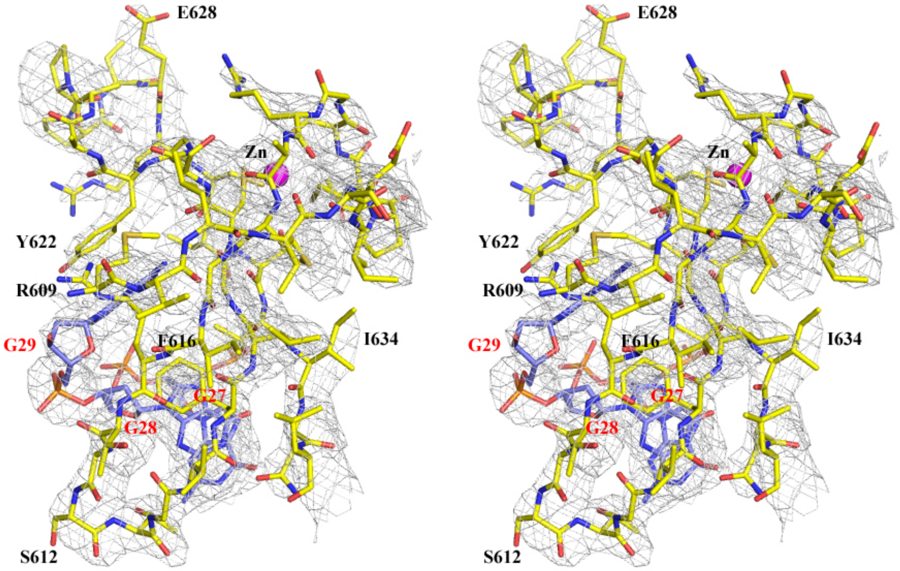

Figure S6. Stereo view of the first C-terminal domain D5 and its interaction with the 3'-end of ssDNA. The domain and its interacting ssDNA are both drawn in stick format. The associated electron density of the domain and the ssDNA is drawn in grey mesh and calculated from a weighted 2Fo-Fc map and contoured at 1σ level.

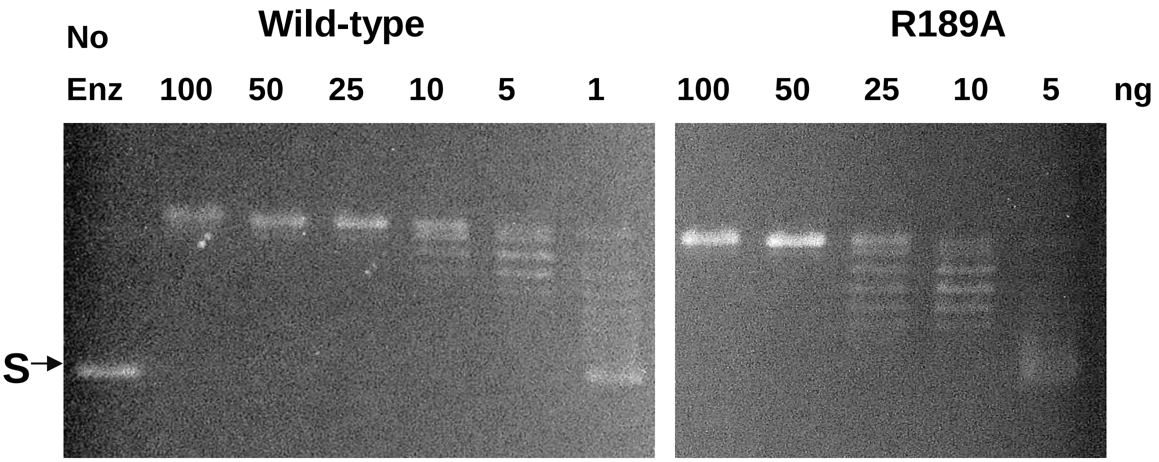

Figure S7. Effect of R189A mutation on EcTOP1 relaxation activity. The indicated amount of wild-type and R189A *E. coli* topoisomerase I was incubated with supercoiled plasmid DNA (S) in buffer with 6 mM $MgCl_2$ to assay the relaxation activity.

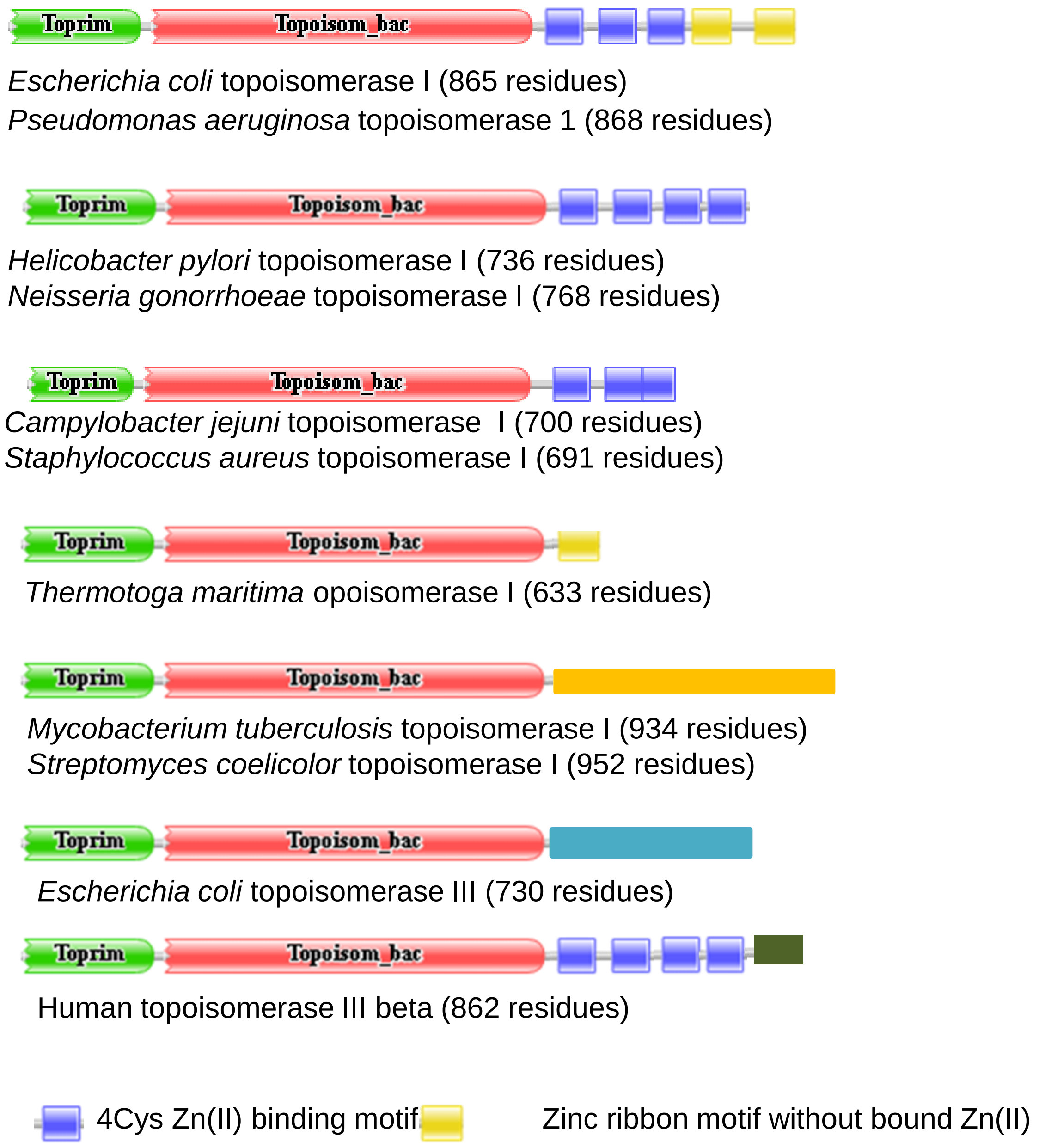

Figure S8. Variations in C-terminal organizations of type IA topoisomerase I and III enzymes. Based in part from domain organization listed for Pfam Topoisom_bac (PF01131).

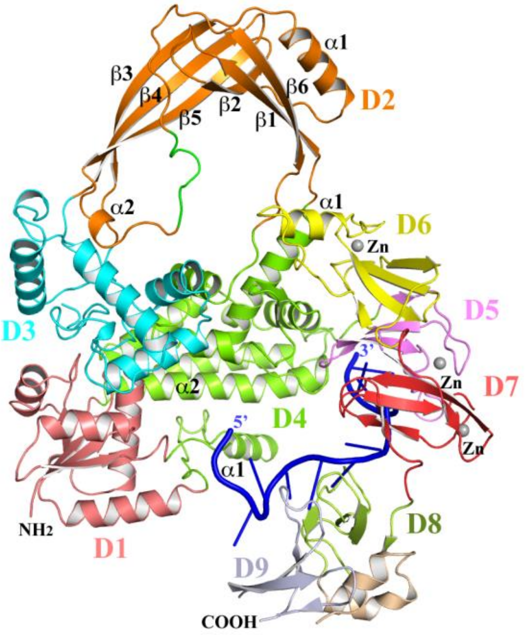

Movie S1. A movie showing a 360-degree rotation (around vertical axis) of full-length EcTOP1 in complex with DNA. The color codes for EcTOP1 domains and ssDNA are the same as in Figure 1B. The Movie is prepared with the program PyMOL (http://www.PyMOL.org).

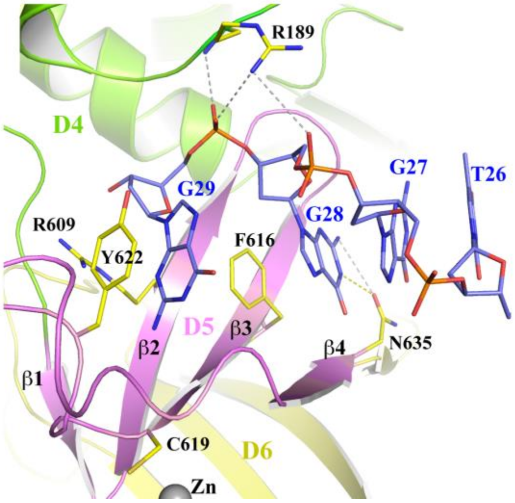

Movie S2. A movie showing a 30-degree rocking (around vertical axis) of EcTOP1 D5 in its interaction with ssDNA. The color codes for different domains in the view and ssDNA are the same as in Figure 4B. The Movie is prepared with the program PyMOL (http://www.PyMOL.org).
